# Simulated functional networks in health and schizophrenia: a graph theoretical approach

**DOI:** 10.1186/1471-2202-12-S1-P63

**Published:** 2011-07-18

**Authors:** Joana Cabral, Etienne Hugues, Gustavo Deco

**Affiliations:** 1 Center for Brain and Cognition, Universitat Pompeu Fabra, Barcelona 08018, Spain; 2 Institut Català de Recerca i Estudis Avançats (ICREA), Barcelona, Spain

## 

In the last decade, particular attention has been paid to the graph theoretical aspects of the brain’s network and its implications in brain function. Small-worldness, path length, efficiency and several other graph theoretical metrics have allowed characterizing anatomical and functional network organization.

In a recent study [1], an analysis of resting-state functional networks of healthy volunteers and people with schizophrenia revealed a number of significant topological differences across groups, namely improved global efficiency, reduced small-worldness, lower clustering, increased hierarchy and greater robustness in patients with schizophrenia compared to healthy subjects. Moreover, these parameters were found correlate with the verbal fluency score, an indicator of disease severity. These results suggest a subtle functional network randomization in schizophrenia. Actually, schizophrenia, characterized by a dysfunctional integration of cognitive processes, is often classified as a disconnection syndrome. This can be understood in terms of abnormal functional connectivity between cortical areas as observed in EEG and fMRI experiments. A disruption of such interactions may therefore underlie the cognitive and behavioral disturbances described in schizophrenia.

In addition, a recent diffusion tensor imaging study [2] reports an overall lower anatomical connectivity in patients than control subjects. This suggests that the functional network alterations underlying schizophrenia could be originated by a widespread deficit in anatomical coupling.

In the present work we investigated the effect of decreasing the neurodynamical coupling strength in the topological properties of simulated functional networks. We used a large-scale dynamical model of local neural ensembles coupled through anatomical white matter fibers. Spontaneous neuronal activity was obtained from simulations and transformed into blood oxygenation level dependent (BOLD) signal. Functional networks were obtained by computing and thresholding the BOLD signals’ correlation matrix. Graph theoretical measures were then calculated for all the simulated graphs.

Results show that the networks’ topological properties vary with coupling strength. In more detail, when the neurodynamical coupling strength is decreased in the model, resulting networks exhibit an increase in global efficiency, hierarchy and robustness and a decrease in small-worldness and clustering, as reported for functional networks of people with schizophrenia. In addition, the shape of the degree distribution also changed accordingly. Moreover, the metrics are in the range of the ones reported experimentally in health and when the coupling strength is decreased by 6%, the measures are closer to the ones reported for schizophrenia (see Table 1).

**Table 1:** Graph theoretical metrics of functional networks in health and schizophrenia

		Global Efficiency	Average Clustering	Hierarchy	Small-Worldness	Robustness to Random Attack	Robustness to Targeted Attack	Degree Distribution		
	
								Variance	Power exponent	Degree cut-off
Health	Exp.	0.744	0.743	0.037	1.61	0.991	0.960	183	3.25	9.45
	
	Sim.	0.688	0.803	0.076	1.72	0.979	0.888	168	3.79	8.17

Schizo.	Exp.	0.746	0.692	0.101	1.53	0.995	0.970	120	6.11	5.37
	
	Sim.	0.697	0.709	0.109	1.51	0.989	0.912	115	4.34	6.40

Exp.: mean experimental value reported in [1]. Sim.: value obtained from simulations. Schizo.: Schizophrenia.

## Conclusions

Graph theoretical alterations of functional networks reported in schizophrenia can be accounted for by a decrease in the neurodynamical coupling strength, in agreement with theories of schizophrenia as a disconnectivity syndrome.
